# Is Theta Burst Stimulation Applied to Visual Cortex Able to Modulate Peripheral Visual Acuity?

**DOI:** 10.1371/journal.pone.0099429

**Published:** 2014-06-10

**Authors:** Sabrina Brückner, Thomas Kammer

**Affiliations:** Department of Psychiatry, University of Ulm, Ulm, Germany; Ecole Polytechnique Federale de Lausanne, Switzerland

## Abstract

Repetitive transcranial magnetic stimulation is usually applied to visual cortex to explore the effects on cortical excitability. Most researchers therefore concentrate on changes of phosphene threshold, rarely on consequences for visual performance. Thus, we investigated peripheral visual acuity in the four quadrants of the visual field using Landolt C optotypes before and after repetitive stimulation of the visual cortex. We applied continuous and intermittend theta burst stimulation with various stimulation intensities (60%, 80%, 100%, 120% of individual phosphene threshold) as well as monophasic and biphasic 1 Hz stimulation, respectively. As an important result, no serious adverse effects were observed. In particular, no seizure was induced, even with theta burst stimulation applied with 120% of individual phosphene threshold. In only one case stimulation was ceased because the subject reported intolerable pain. Baseline visual acuity decreased over sessions, indicating a continuous training effect. Unexpectedly, none of the applied transcranial magnetic stimulation protocols had an effect on performance: no change in visual acuity was found in any of the four quadrants of the visual field. Binocular viewing as well as the use of peripheral instead of foveal presentation of the stimuli might have contributed to this result. Furthermore, intraindividual variability could have masked the TMS- induced effects on visual acuity.

## Introduction

Repetitive transcranial magnetic stimulation (rTMS) is a noninvasive technique to induce a virtual lesion in a cortical area, allowing the investigation of cortical functions. Primarily used in the motor system [1], the technique was successfully transferred to other cortical regions, i.e. Wernicke's area [2], parietal lobes [3] or left dorsolateral prefrontal cortex [4] in order to specifically interfere with the function of the given area. In the visual system, rTMS protocols are often applied either to occipito-lateral sites targeting V5/MT^+^ or more medial sites at the occipital pole. For the latter ones in most of the studies the exact stimulation site (hot-spot) is defined by an individual physiological reaction, the perception of TMS-induced light flashes, so called phosphenes. They can be elicited from a quite large area over the occipital pole. In most of the cases TMS reaches a mixture of V1, V2 and V3 [5]. An exclusive stimulation of a certain visual area (e.g. V1) has been shown to be possible only in selected subjects [6]. In most of the studies applying rTMS to the visual cortex phosphene thresholds (PTs) are the dependent variable [7]–[9]. PT measurement indeed is the most common tool to explore the after-effects of rTMS protocols applied to the visual cortex, but it just points to a change of excitability per se and not to any functional consequences for visual performance. Thus, it is of interest to investigate the modulation of visual cortex excitability by different behavioral experiments. For example, in one study static contrast sensitivity (sCS) was evaluated before, during, immediately after and 10 minutes after both monophasic and biphasic 1 Hz rTMS applied to the occipital cortex [10]. With a vertical coil position decreased sCS was found only after monophasic stimulation with induced currents upwards. Reversed currents and biphasic stimulation in both directions showed no significant effects. It was concluded that primary visual functions such as contrast detection can be altered by rTMS. Another study [11] suggested an inhibitory effect of low-frequency rTMS on optic flow perception.

In the motor system theta burst stimulation (TBS, 3 pulses at 50 Hz bursting every 200 ms, resulting in 5 Hz burst pattern) strongly modulated cortical excitability depending on the grouping of the pattern [12]. In this initial study, continuous TBS (cTBS, 600 pulses continuously bursting over a period of 40 s) inhibited the motor cortex, while intermittent TBS (iTBS, every 10 bursts a pause of 1.8 s occurs, 600 pulses in total) resulted in an increase in motor cortex excitability. Although subsequent studies obtained reversed effects using modified TBS paradigms [13]–[15], most researchers replicated the initially observed results in the motor system (e.g. [13]–[19]). Compared to conventional 1 Hz rTMS protocols, inhibition in the same range was reached by cTBS with lower stimulation intensities and much shorter application time. Since cTBS is more convenient for subjects it was suggested that cTBS might replace 1 Hz rTMS. For instance, both 1 Hz rTMS and cTBS applied to the frontal eye field had inhibitory effects on saccade triggering [20]. Prolonged reaction times in a lexical decision task were observed following cTBS and 1 Hz rTMS applied to left the superior temporal cortex, respectively [21].

In the visual system PTs increased after 1 Hz rTMS [9] as well as after cTBS [22]. Expecting a virtual lesion effect, the impact of 1 Hz rTMS and cTBS on different visual psychophysical tasks were investigated [23]. However, an improvement in discrimination of some visual features were found, depending on the type of stimulation and task. This finding demonstrates that the direction of phosphene modulation does not distinctly predict the change in a behavioral task.

To further analyze the effects of rTMS on visual perception we tested peripheral visual acuity following several rTMS pattern. We applied continuous (cTBS) and intermittend theta burst stimulation (iTBS) with stimulation intensities ranging between 60% to 120% of individual PT, as well as monophasic and biphasic 1 Hz rTMS at PT intensity. Due to the retinotopic organization of the visual cortex, we tested visual acuity in each quadrant of the visual field.

## Methods

### 1. Subjects

In total 34 subjects participated in the study: 12 in experiment 1 (mean age 24.2±2.3 years, 5 men) and 12 in experiment 2 (mean age 24.8±2.8 years, 5 men). Experiment 3 included 15 subjects (mean age 24.9±2.6 years, 6 men), who took also part in either experiment 1 or experiment 2. After analyzing the results, we started two replication tests (see results section). The first replication test comprised 9 subjects (mean age 25.4±1.7 years, 4 men) who had already participated in experiment 1 or 2. Ten experiment-naïve subjects (mean age 24.0±3.5 years, 2 men) participated in the second replication test. All but one participant was right- handed and all had normal or corrected-to-normal visual acuity. Exclusion criteria were metallic implants, major medical illness, prior history of neurological or psychiatric disorders, in particular a history of epileptic fits, chronic tinnitus, drug abuse or alcoholism, any medication with the exception of oral contraceptives. All participants gave their written informed consent for the experiments and were paid for participation. The study followed the Declaration of Helsinki and was approved by the ethics committee of the university of Ulm.

### 2. Setup and task

Subjects sat in a comfortable chair while they were stimulated with a Magpro X100 stimulator (MagVenture Farum, Denmark), using a figure-of-eight-coil, MC-B70, and biphasic pulses (with the exception of experiment 3). The coil position in relation to the head was monitored and registered with the frameless stereotactic positioning system BrainView (V2, Fraunhofer IPA, Stuttgart, Germany, cf. [24]).

**Figure 1:**
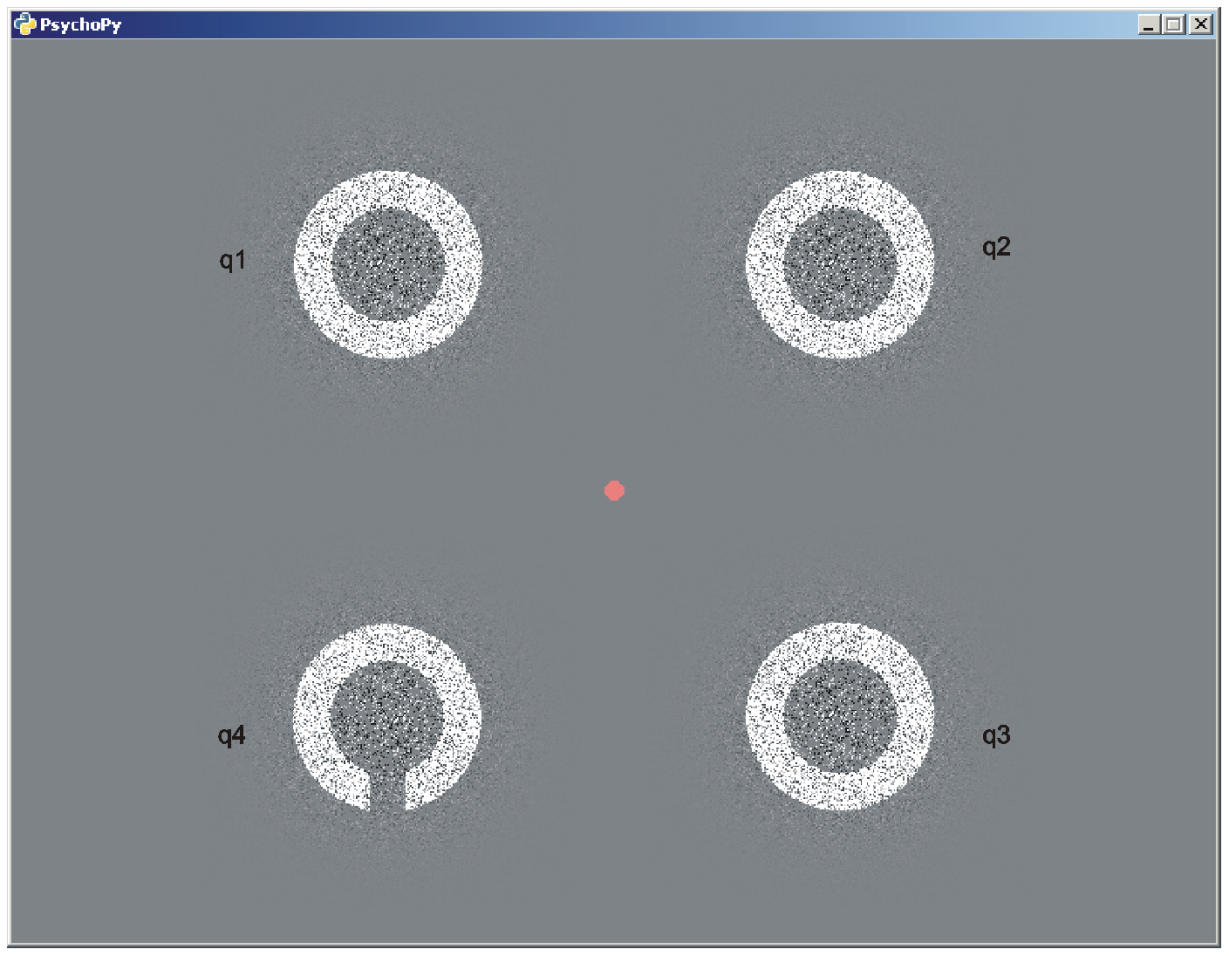
Layout of the task. The four quadrants of the visual field are labeled as q1, q2, q3, and q4. For sake of clarity, eccentricity and size of the stimuli are modified.

The visual task consisted of a simultaneous acuity measurement in the four quadrants of the visual field. It was realized in PsychPy (v. 1.70, [25]) on a standard personal computer and a cathode ray tube computer monitor (21″, iiyama Vision Master Pro 500, iiyama, Tokyo, Japan) with a framerate of 100 Hz and a resolution of 1024×768 pixel. Four rings of the same diameter were presented simultaneously in the four quadrants at a distance of 663.1′ (10.6°) from fixation point each. Three rings were closed, and one ring had a gap (Landolt C optotype) oriented either up, right, down, or left. The rings were displayed on a full pixel base. No anti-aliasing was applied. The rings were displayed for 100 ms followed by a mask consisting of random noise pixels. To raise complicacy, during ring presentation some noise pixels were added (Fig. 1). The quadrant containing the Landolt C was at random from trial to trial. Subjects had to report the direction of the gap only and should neglect the quadrant of presentation (four alternative forced choice task). In order to determine acuity threshold the size of the Landolt C as well as the diameter of the closed rings was varied for each quadrant separately following a simple 2∶1 staircase procedure. The variation occurred in steps of one pixel (0.0375° or 2′15″). Thus, four independent staircase procedures were randomly interleaved in each run. The run was terminated after at least 7 reversals in each staircase. To provide random presentation of the Landolt C in all quadrants, all staircases just continued running until the last one reached the 7^th^ reversal. In order to motivate subjects in each staircase bonus trials were interleaved every 5^th^ to 9^th^ presentation, where the diameter of the rings was 5 pixels larger with respect to the actual staircase value.

Acuity thresholds were estimated for each run and quadrant separately applying a sigmoidal fit to the observed response data generated by the staircase procedure (psignifit, [26]). Visual acuity threshold was defined as the reversal point of the logistic function (62.5% correct answers).

### 3. Experimental design

Subjects always started with a familiarization session. Then, several sessions followed on separate days each in order to investigate the effect of the different TMS protocols. In the familiarization session subjects trained to observe phosphenes and to run the behavioral task. Phosphene perception had to fulfill the following criteria (cf. [5]): a) dependence on the stimulated hemisphere, i.e. perception in the left visual field with stimulation at the right occipital pole and vice versa [27]; b) visibility with both states, eyes open or closed [28]; c) dependence on gaze direction [27]. Phosphene perception thresholds from the left occipital pole were measured following a previously established protocol [29]. First, the stimulation site was determined by moving the coil in steps of about 5 mm over the left occipital pole while the subject was stimulated with suprathreshold intensity known from the familiarization procedure until he or she observed a sharply delineated phosphene clearly restricted to the right visual field (“hot-spot”). Then the coil was rotated in order to determine the current direction inducing the strongest percept [5]. In most of the cases this was a latero-medial direction of the induced current. Then, 49 magnetic stimuli were delivered at 7 different stimulator output intensities in steps of 3%, with intensities randomly intermixed (method of constant stimuli). The experimenter released the magnetic stimulation manually at a frequency below 0.25 Hz, and the subject reported verbally the presence or absence of a phosphene after each stimulus (“Yes” or “No”). A sigmoidal fit applied to the subject's responses generated the individual PT (reversal point of the logistic function, 50% correct answers). To get familiar with the visual acuity task, subjects were trained in a practice session with 5 levels of increasing difficulty. Then subjects passed 6 additional training trials.

In the first rTMS session on a separate day the procedure to determine a phosphene hot-spot was repeated and the optimum position was registered with the neuronavigation system with respect to anatomical landmarks of the skull. Then PT measurement was repeated to determine the reference threshold value.

In all rTMS sessions, prior to repetitive stimulation subjects first had to complete 4 trials of the visual acuity task. While the first 2 trials were discharged (training trials), the average of trial 3 and 4 was taken as baseline visual acuity (pre stimulation). Then rTMS was applied over the predetermined phosphene hot-spot. Post stimulation visual acuity was measured immediately after rTMS and then every 10 minutes over a period of 40 minutes after stimulation, i.e. 5 times in total.

Since phosphenes evoked with single pulse TMS over occipital cortex usually appear in the contralateral lower quadrant of the visual field [5], we decided to apply TMS always to the left hemisphere. Thus, all subjects perceived phosphenes in the lower right quadrant (q3). Due to the retinotopic organization of the visual cortex, we expected a modulation of visual acuity threshold particularly in this part of the visual field. To explore whether a modulation of visual acuity threshold would occur particularly in the corresponding part, we measured visual acuity of the four quadrants separately.

To prevent carry-over effects and to make sure that subjects kept familiar to the behavioral task, the time between two sessions was 1 to 8 days.

#### 3.1. Experiment 1: cTBS

The subjects in experiment 1 were repetitively stimulated with cTBS (bursts of 3 pulses at 50 Hz, every 200 ms) for a total of 600 pulses. Stimulation intensities were 60%, 80%, 100% and 120% of individual PT. In order to control for putative carry-over effects, the first intensity was either 60% or 120%, alternating across subjects sequentially. In the following three rTMS sessions intensity was either increased up to 120% or decreased down to 60%, respectively.

#### 3.2. Experiment 2: iTBS

Experiment 2 was similar to experiment 1, but iTBS (10 bursts of 3 pulses at 50 Hz, every 200 ms, 20 repetitions every 10 s) was applied for a total of 600 pulses. None of the subjects in experiment 2 had already participated in experiment 1.

#### 3.3. Experiment 3: 1 Hz rTMS

In this experiment repetitive stimulation consists of 1 Hz rTMS applied with 100% of individual PT, both with monophasic and biphasic pulses for 15 min each. Subjects (n = 15) were a subgroup of participants from experiment 1 and experiment 2. The 1 Hz sessions were scheduled after the 4 TBS sessions. Thus, subjects who took part in this experiment had to complete six sessions. Stimulation protocol was alternating across subjects. All other parameters were similar to those in the other two experiments.

### 4. Data analysis

Baseline visual acuity values of the subjects who underwent 6 rTMS sessions were analyzed with respect to the number of session to explore possible learning effects.

Change of visual acuity was defined as the difference of pre-stimulation acuity minus post-stimulation acuity. Data were submitted to repeated-measures analysis of variance (rmANOVA) for each experiment (Statistica V. 10, StatSoft GmbH, Hamburg, Germany). Sphericity requirements for the rmANOVAs were assessed by using Mauchly's test. In case of violation, Greenhouse-Geisser correction was applied and *ε* values are reported together with the corrected *p* values. Post-hoc analyses were performed using t-tests.

Raw visual acuity data are presented as supplementary material (Table S1).

## Results

All but one subject tolerated rTMS well and no serious adverse effects occured. When iTBS in experiment 2 was applied with 120% of individual PT, stimulation was stopped for one subject because this intensity was too painful. In two other subjects, we failed to apply iTBS at 120% PT. In one case, stimulation was aborted due to overheating of the coil. In the other case the subject skipped the whole session for lack of time. Therefore, we did not include the data of the 120% sessions in the analysis of experiment 2.

Baseline visual acuity values of the 15 subjects with 6 baseline measurements each (experiment 1+3 or 2+3) were subjected to an rmANOVA with the factors SESSION (1, 2, 3, 4, 5, 6) and QUADRANT (q1, q2, q3, q4). Significant main effects were found for both factors QUADRANT (*F*_(3,42)_ = 4.58, *p* = 0.017, *ε* = 0.71) and SESSION (*F*_(5,70)_ = 3.13, *p* = 0.013), but no interaction. Mean baseline visual acuity threshold was lowest in the upper right (q2) and highest in the lower left (q4) part of the visual field (q1: 0.260°, q2: 0.250°, q3: 0.261°, q4: 0.282°), decreasing continuously from the first session (0.274°) to the last session (0.254°).

### 1. Experiment 1: cTBS

**Figure 2:**
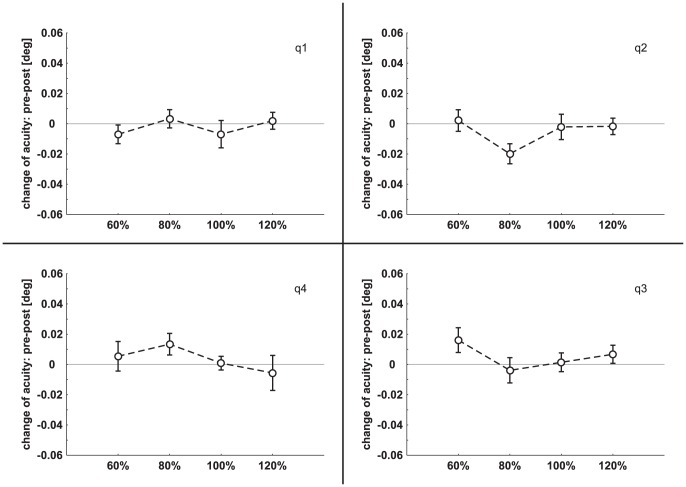
Change of visual acuity (mean ± SEM) in the four quadrants following cTBS. On the abscissa the four cTBS intensities are depicted. The four quadrants of the visual field are labeled as q1, q2, q3, and q4. For sake of clarity values over time are averaged.

Mean PT value was 38.6%±8.9% (range 26%–50%) of maximum stimulator output. Pre minus post visual acuity data were subjected to an rmANOVA with the factors INTENSITY (60%, 80%, 100%, 120%), QUADRANT (q1, q2, q3, q4) and TIME (0, 10, 20, 30, 40 minutes post stimulation). No significant main effect was found for any factor (INTENSITY: *F*_(3,33)_ = 0.44, *p* = 0.73; QUADRANT: *F*_(3,33)_ = 1.61, *p* = 0.20; TIME: *F*_(4,44)_ = 1.04, *p* = 0.40) and there was no interaction. In Fig. 2 data of experiment 1 (pre minus post visual acuity) are shown separately for each quadrant.

### 2. Experiment 2: iTBS

**Figure 3:**
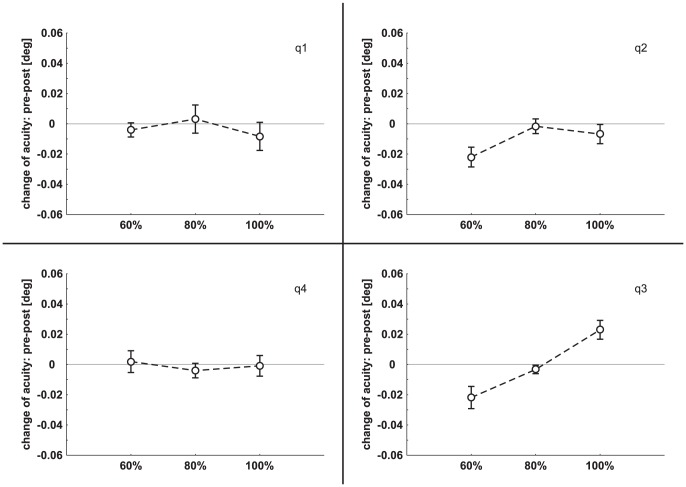
Change of visual acuity (mean ± SEM) in the four quadrants following iTBS. On the abscissa the three iTBS intensities are depicted. The four quadrants of the visual field are labeled as q1, q2, q3, and q4. For sake of clarity values over time are averaged.

Mean PT value was 42.0%±9.7% (range 28%–55%) of maximum stimulator output. As is experiment 1, pre minus post visual acuity data were subjected to an rmANOVA with the factors INTENSITY (60%, 80%, 100%), QUADRANT (q1, q2, q3, q4) and TIME (0, 10, 20, 30, 40 minutes post stimulation). No significant main effect was found for any factor (INTENSITY: *F*_(2,22)_ = 2.70, *p* = 0.09; QUADRANT: *F*_(3,33)_ = 1.65, *p* = 0.20; TIME: *F*_(4,44)_ = 0.35, *p* = 0.84), but the interaction of the three factors was significant (*F*_(24,264)_ = 1.68, *p* = 0.027) as well as the interaction of the factors intensity and quadrant (*F*_(6,66)_ = 4.38, *p* = 0.001). Post-hoc analysis of the intensity-quadrant-interaction revealed a decrease of visual acuity in the critical quadrant (lower right quadrant, q3) after stimulation applied with 60% of individual PT (*t*_(12)_ = 2.99, *p* = 0.012) and an increase after application of 100% intensity (*t*_(12)_ = 3.71, *p* = 0.003). Fig. 3 shows pre minus post visual acuity for each quadrant separately.

### 3. Experiment 3: 1 Hz rTMS

**Figure 4:**
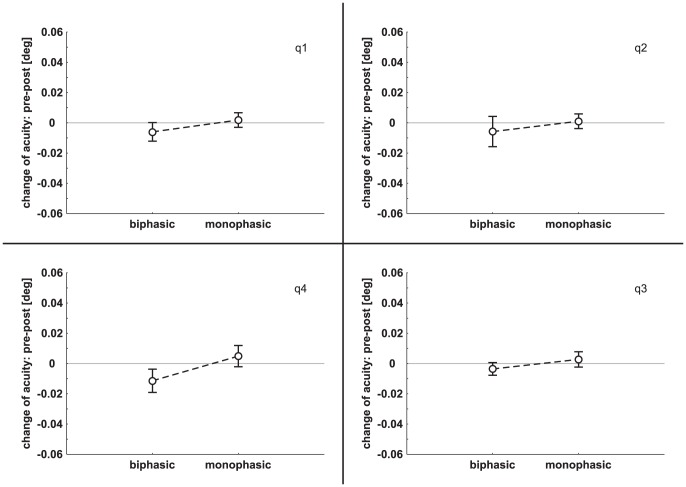
Change of visual acuity (mean ± SEM) in the four quadrants following 1 Hz rTMS. On the abscissa the two 1

Mean PT value was 39.6%±8.5% (range 28%–50%) of maximum stimulator output for biphasic and 54.2%±15.0% (range 35%–75%) for monophasic pulses. An rmANOVA with the factors PULSE (biphasic, monophasic), QUADRANT (q1, q2, q3, q4) and TIME (0, 10, 20, 30, 40 minutes post stimulation) of the 1 Hz data revealed no significant main effect (PULSE: *F*_(1,14)_ = 3.99, *p* = 0.07; QUADRANT: *F*_(3,42)_ = 0.08, *p* = 0.97; TIME: *F*_(4,56)_ = 1.39, *p* = 0.25) and no interaction. In Fig. 4 pre minus post visual acuity of experiment 3 is shown separately for each quadrant.

### 4. Replication test 1: iTBS 100%

Based on the significant increase of visual acuity in the right lower quadrant of the visual field after iTBS application with 100% of individual PT in experiment 2 we tried to replicate this result. Nine subjects who had taken part in one of the other experiments before were again stimulated with iTBS 100% intensity. Visual acuity was measured prior to stimulation as described before and once directly after iTBS.

**Figure 5:**
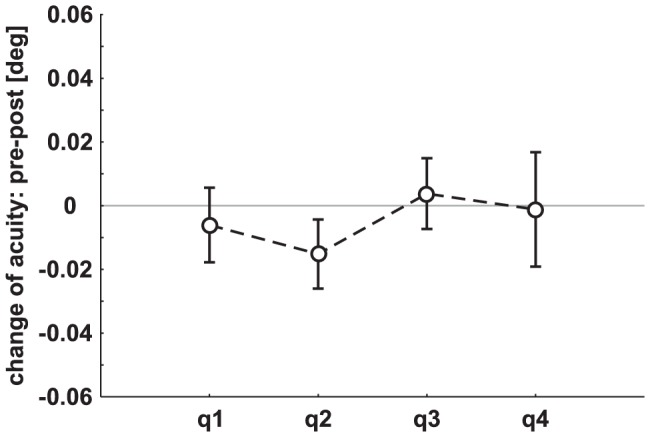
Change of visual acuity (mean ± SEM) in the four quadrants following cTBS at 100% PT(replication test 1).

Mean PT value was 40.2%±8.4% (range 33%–49%) of maximum stimulator output. Data were subjected to an rmANOVA with the factor QUADRANT (q1, q2, q3, q4), and no significant effect was found (*F*_(3,24)_ = 0.37, *p* = 0.78), thus the observed effect could not be replicated (see Fig. 5).

### 5. Replication test 2: iTBS 40%, 60%

**Figure 6:**
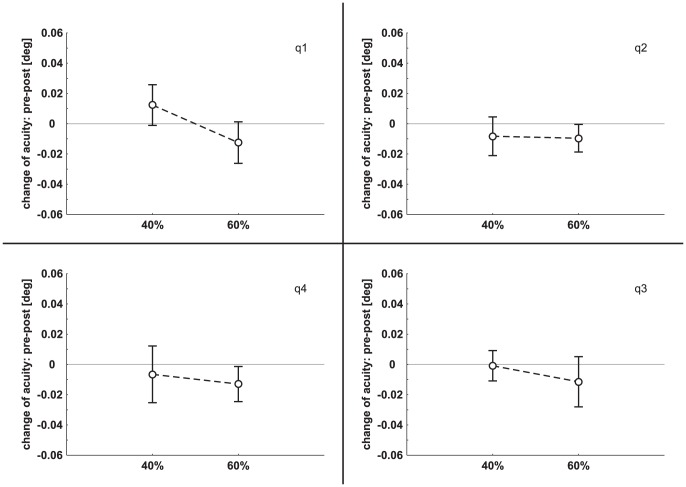
Change of visual acuity (mean ± SEM) in the four quadrants following iTBS at 40% and 60% PT (replication test 2). On the abscissa the two iTBS intensities are depicted. The four quadrants of the visual field are labeled as q1, q2, q3, and q4.

Based on the significant decrease of visual acuity in the right lower quadrant of the visual field after iTBS application with 60% of individual phosphene threshold in experiment 2 we tried to replicate this result. Ten subjects who had not taken part in one of the previously described experiments before were trained on the visual acuity test as described in the methods section. In two sessions, subjects were stimulated with 40% and 60% iTBS intensity, respectively. Visual acuity was measured prior to stimulation similar to the other experiments and once directly after stimulation. Mean PT value was 29.9%±7.2% (range 18%–44%) of maximum stimulator output. Data were subjected to a rmANOVA with the factors INTENSITY (40%, 60%) and QUADRANT (q1, q2, q3, q4). There was no significant main effect (INTENSITY: *F*_(1,9)_ = 0.74, *p* = 0.41; QUADRANT: *F*_(3,27)_ = 0.27, *p* = 0.84) and no interaction (*F*_(3,27)_ = 0.26, *p* = 0.86). Thus, second replication test failed as well. In Fig. 6 pre minus post visual acuity of replication test 2 are shown separately for each quadrant.

## Discussion

To explore the effects of rTMS on visual perception, we measured peripheral visual acuity before and after several stimulation protocols and intensities. In contrast to our hypothesis neither cTBS nor iTBS at any stimulation intensity modulated visual acuity measured in the four quadrants of the visual field. A control experiment with monophasic as well as biphasic 1 Hz rTMS showed also no modulation of visual acuity. Although we first observed an increase of visual acuity with iTBS 100% and a decrease with 60% PT intensity in the critical quadrant of the visual field (experiment 2), we were not able to replicate the results.

In first attempts to modulate visual cortex function, conventional 1 Hz rTMS was applied to the occipital pole. In several studies an increase of PT was demonstrated following stimulation [7]–[9]. Exploring perceptual changes after the application of 1 Hz rTMS [10], a decrease in static contrast sensitivity after 10 min of monophasic stimulation, but no effect after biphasic stimulation was found. The effects of the theta burst protocol were investigated in the visual system for both PTs and perceptual tasks. Increased PTs were found after cTBS but not after iTBS, both applied with 80% of individual PT [22]. In another study, both cTBS and rTMS at 1 Hz improved discrimination in 6 out of 7 and 4 out of 5 subjects in two different coarse orientation tasks, respectively [23]. No effect on fine orientation discrimination tasks was observed. This suggests that rTMS effects in the visual cortex depend not only on parameters such as intensity or stimulation protocol, but also on the type of the task. One possible explanation for the absence of a significant change of visual acuity in our experiment is that our task might not be sensitive enough for the rTMS induced modulations. Intraindividual variability of measured visual acuity was quite high and could have masked the TMS- induced effect. One might ask whether a mixture of attention and learning contributes to the variability observed. In our experiment, baseline of peripheral visual acuity was 0.272° (mean), which is in line with previous findings showing a visual acuity of about 0.2° with ∼10° eccentricity (i.e. [30], [31]). Comparing baseline visual acuity in the chronology of sessions, we detected a small and continuous increase of performance, indicating a training effect. Previous findings [32] showed some evidence for fast perceptual learning, not consistent over subjects and tasks. This suggests that learning in peripheral visual acuity may depend on the task as well as on the subject per se. However, high test-retest-variability within subjects is often reported for peripheral visual acuity and it is supposed that variability increases with lower visual acuity [33]. Nevertheless, since we controlled for possible learning effects and carry-over effects by alternating the different sessions across subjects, it is unlikely that learning masked modulatory effects caused by rTMS.

Using a visual detection task, the influence of selective attention on effects of iTBS applied to the motor cortex was investigated [34]. LTP- like effects in low-load conditions were found, but no effects under high-attentional load were reported, indicating that attention can be a modulator of cortical changes as well. Supposing that our visual task requires high attention levels due to the short duration of stimulus presentation and the high eccentricity, this could have eliminated the TMS-induced effects on visual acuity. Presentation time was short (100 ms) to prevent saccades, which are known to have a minimum latency of about 150 ms [35]. A possible strategy to reduce the level of attention might be decreasing eccentricity or using foveal stimuli for visual acuity measurements. However, in such a task it would not be possible to distinguish between the quadrants of the visual field. A putative TMS-effect then could not be assigned to a particular field of the visual cortex.

A possible limitation of our study is the lack of using an eye-movement monitoring system. Measurement of peripheral visual acuity requires a stable and correct fixation. We did not control for eye movements by measurement, but subjects were trained to maintain fixation. Since the occurrence of peripheral targets was counterbalanced and randomized, the best strategy to observe targets was keeping fixation in the middle of the screen.

Our task consisted of four independent acuity measurements in the four quadrants of the visual field (10.6° eccentricity), respectively, that are intermixed. Hence, stimuli were always presented in the periphery to investigate whether there is a quadrant- specific effect of offline rTMS. We expected effects in particular in the periphery of the visual field for the following reasons: a) Phosphenes evoked with single pulse TMS over occipital cortex usually appear in the contralateral lower quadrant and often exclude foveal parts [5]. b) visual suppression following triggered single pulse TMS also occurs mainly in parafoveal and peripheral parts of the contralateral visual field [36]–[38]. Since we determined the phosphene hot-spot always in the left hemisphere, we expected rTMS effects in the right lower part of the visual field. In our former studies, we observed a retinotopic relation between phosphenes and visual extinction measured by means of static perimetry [5], [39]. However, phosphenes and scotomas are just consequences of a basal excitation of the visual cortex. The successful detection of the orientation of an optotype might require more complex visual functions in basal and higher visual cortical areas.

Perception of changes in naturalistic scenes is more difficult for peripheral than for foveal viewing [40], and visual enumeration decreases with eccentric viewing [41]. Thus, according to our data it is conceivable that uncertainty in the periphery may result in more variability in visual acuity. Applying transcranial direct current stimulation (tDCS) to the occipital cortex, a significant increase in contrast sensitivity following anodal stimulation was observed only for central positions, but not for positions in the periphery [42]. It was argued that due to the larger representation of the fovea as well as its location closer to the skull it is more susceptible to modulatory effects. Hence, one could infer that TMS effects might be less pronounced for peripheral representations since field strength decay with increasing distance to the coil and cortical representation of peripheral visual field located in deeper parts of the occipital cortex receive less TMS energy. This would explain the absence of modulatory effects of rTMS in our data. However, this is in contrast to the observation that TMS induced phosphenes and scotomas occur more pronounced in the periphery of the visual field rather than in foveal regions [5], [36]–[38]. Assuming that not only V1 but predominantly higher visual areas such as V2d and V3 are targeted by the TMS pulse, representations of peripheral visual field are located close to the skull, too [5], [38].

Another conceivable explanation for our results might be that subjects performed our task binocularly. As suggested earlier [43], this might protect against the TMS-effects on a visual perceptual task because the cortical representation of the stimuli is more robust [44] compared to monocular viewing.

Finally, we cannot exclude an interaction between the persistence of a modulatory rTMS effect and the activity of the visual system due to the acuity task. In the motor cortex, the contraction of a muscle belonging to the stimulated area can reverse the TBS after-effect [13]. Furthermore, it might be worthwhile to distinguish between threshold change and suprathreshold modulation, since effects on both parameters might be different following rTMS [45]. Therefore, it would have been advantageous to measure PT following rTMS, too. However, according to previous reports [7]–[9] we assumed a clear modulation of phosphene thresholds after 1 Hz rTMS. To our knowledge, modulatory effects of cTBS on PT have been shown in the literature in a single study [22]. Thus, a PT measurement following TBS would have been informative. Nevertheless, because the effects of rTMS protocols on behaviour are reported to be short-lasting [46], we decided to restrict our measurements to visual acuity.

In summary, it might be likely that the used experimental procedure is not sensitive to observe the expected effects of rTMS on visual perception, although the task was sensitive enough to detect small continuous training effects. To further investigate the consequences of different rTMS paradigms applied to the visual cortex one should consider the present limitations. On the one hand, the use of foveal stimuli and monocular vision are recommended. On the other hand, it might be advantageous to investigate the effect of rTMS on PT before and after the visual acuity task to examine whether the task itself protects against changes in cortical excitability.

Although no behavioral effect was observed, the systematic application of TBS at higher stimulation intensities is of certain value. Usually, TBS is applied with 80–90% of active motor threshold [47]. To our knowledge, up to now the highest TBS intensity applied was 100% of resting motor threshold (RMT; i.e. [48], [49]) using a modified cTBS protocol [50]. Although motor- and phosphene thresholds are not correlated, in a within-design PTs were shown to be higher compared to RMTs [51]. In the present study, the application of cTBS and iTBS to the visual cortex in a wide range of stimulation intensities from 60% to 120% of individual PT was well tolerated by the subjects. In contrast to a recent report of a seizure caused in a healthy subject when applying 150

pulses of cTBS to the left motor cortex with 100% of individual RMT [52], no serious adverse effects were noticed in our study. Although the stimulation intensities we used were higher than those usually applied [47], in only one case we had to abort the stimulation (iTBS, 120% PT) due to intolerable pain. In 21 subjects, high-intensity TBS (120% PT) was applied without adverse effects. Since TBS with higher stimulation intensity might result in more pronounced effects [21], the absence of adverse effects in this study might encourage researchers to test TBS in higher intensity ranges.

## Supporting Information

Table S1 **Raw data (visual acuity thresholds).** Legend is included in the file.
(XLS)
